# Short- and Long-Term Effects of Prenatal Exposure to Iron Oxide Nanoparticles: Influence of Surface Charge and Dose on Developmental and Reproductive Toxicity

**DOI:** 10.3390/ijms161226231

**Published:** 2015-12-18

**Authors:** Kristin R. Di Bona, Yaolin Xu, Marquita Gray, Douglas Fair, Hunter Hayles, Luckie Milad, Alex Montes, Jennifer Sherwood, Yuping Bao, Jane F. Rasco

**Affiliations:** 1 Department of Biological Sciences, the University of Alabama, Tuscaloosa, AL 35487, USA; msgray1@crimson.ua.edu (M.G.); dcfair@crimson.ua.edu (D.F.); hshayles@uab.edu (H.H.); lemilad@crimson.ua.edu (L.M.); amontes2784@gmail.com (A.M.); jrasco@ua.edu (J.F.R.); 2 Department of Chemical and Biological Engineering, the University of Alabama, Tuscaloosa, AL 35487, USA; yxu22@crimson.ua.edu (Y.X.); jasherwood@crimson.ua.edu (J.S.); ybao@eng.ua.edu (Y.B.)

**Keywords:** iron oxide, nanoparticles, developmental toxicity, fetotoxicity, surface charge, nanotoxicity

## Abstract

Iron oxide nanoparticles (NPs) are commonly utilized for biomedical, industrial, and commercial applications due to their unique properties and potential biocompatibility. However, little is known about how exposure to iron oxide NPs may affect susceptible populations such as pregnant women and developing fetuses. To examine the influence of NP surface-charge and dose on the developmental toxicity of iron oxide NPs, Crl:CD1(ICR) (CD-1) mice were exposed to a single, low (10 mg/kg) or high (100 mg/kg) dose of positively-charged polyethyleneimine-Fe_2_O_3_-NPs (PEI-NPs), or negatively-charged poly(acrylic acid)-Fe_2_O_3_-NPs (PAA-NPs) during critical windows of organogenesis (gestation day (GD) 8, 9, or 10). A low dose of NPs, regardless of charge, did not induce toxicity. However, a high exposure led to charge-dependent fetal loss as well as morphological alterations of the uteri (both charges) and testes (positive only) of surviving offspring. Positively-charged PEI-NPs given later in organogenesis resulted in a combination of short-term fetal loss (42%) and long-term alterations in reproduction, including increased fetal loss for second generation matings (mice exposed *in utero*). Alternatively, negatively-charged PAA-NPs induced fetal loss (22%) earlier in organogenesis to a lesser degree than PEI-NPs with only mild alterations in offspring uterine histology observed in the long-term.

## 1. Introduction

Over the past few decades, interest, design, and implementation of engineered nanomaterials have grown exponentially, and the resulting influx of nanomaterial-containing products has greatly increased human exposure [1,2]. Engineered nanomaterials are defined as intentionally produced materials with at least one dimension ranging from 1 to 100 nm [3]. Nanomaterials, including nanoparticles (NPs), exhibit physical and chemical properties unique from larger materials with the same chemical composition, due primarily to their small size and high surface to volume ratios [3]. Due to these unique properties, NPs are now routinely employed in many fields including industrial applications (e.g., pigments, catalysts, and solar cells), biomedical applications (e.g., drug delivery and diagnostics), and consumer products (e.g., sunscreens, paints, and food packaging) [4,5,6,7]. 

Magnetic NPs are of particular interest to the field of biomedicine due in part to their ability to be manipulated using a magnetic field and directed to a particular tissue of interest, as well as for their use as a contrast agent in magnetic resonance imaging (MRI) (Valdiglesias *et al.* [3]) [8]. Magnetic NPs with an iron oxide core have been investigated for many biomedical applications such as drug delivery [9,10] and are currently clinically approved to treat anemia induced by chronic kidney disease (Gupta [11]) [8,12,13]. Biomedical investigations have even led to the use of iron oxide NPs in several clinical trials [8,12,13]. In addition to use in biomedicine, iron oxide NPs are now routinely used in environmental remediation applications at more than 50 sites in the United States, resulting in a greater risk of human exposures [14,15,16,17,18]. Iron oxide NPs are generally thought to be safe and “biocompatible,” primarily through the use of cell viability assays and the potential for NPs to utilize typical iron metabolic pathways, though the mechanisms are not well understood [12]. Greater evaluation and understanding of the mechanisms of iron oxide nanotoxicity are required for the future design and implementation of iron oxide NPs resulting in human exposures.

The basic properties of NPs, which make them appealing for many industrial, biomedical, and consumer applications, also present unique toxicological hazards [19]. The inherent small size of NPs enables greater absorption and potentially greater bioavailability than bulk materials. Greater bioavailability of NPs can be beneficial for biomedical uses, but may lead to enhanced toxicity (e.g., crossing the placenta). Various studies have investigated both *in vitro* and *in vivo* toxicity of NPs. *In vitro*, NPs have demonstrated the ability to induce oxidative stress, damage DNA and mitochondria, and disrupt gene expression, while *in vivo* NPs have been shown to induce inflammation or suppress the immune response [3,20,21]. Though little is known about the mechanisms of NP toxicity, various factors have been shown to influence observed toxicities, such as core material, size, shape, surface functionalization, and surface charge [3,19,21].

Reported studies investigating the potential toxicity of NPs primarily focus either *in vitro* or *in vivo* on adult, healthy models, with little or no focus on susceptible populations such as pregnant women and the developing fetus. Pregnant women are particularly susceptible to exogenous stimuli including NPs, and the small size of the NPs negates the size-selectivity of the placental barrier, which protects the embryo from exogenous toxins [22,23]. Exposure to the developing fetus can be devastating due to inherent increased susceptibility to environmental toxins [22,24,25]. *In utero* exposures can also lead to long-term developmental toxicity that may be observed later in life. For example, trans-placental transfer has been observed with titanium dioxide NPs leading to brain and nervous system damage as well as with carbon NPs resulting in reduced sperm production in male offspring [26,27]. Mice exposed to platinum NPs demonstrated potential long-term effects of gestational NP exposure. While there were no differences in fetal death or NP accumulation in the pups, decreased growth rates and pup mortality were significantly increased in those exposed to platinum NPs *in utero* [28]. Additional animal studies performed using several different metal NPs further illuminate the potential harmful effects of NP exposure during pregnancy (Li [20]).

Though exposure to iron oxide NPs is generally believed to be safe, concerns remain about increased exposure to susceptible populations, such as pregnant women and the developing fetus [20]. Previous research by our lab has demonstrated the ability of 12 nm Fe_2_O_3_-NPs (hydrodynamic size ~28–30 nm with polymer coatings) to cross the placenta and accumulate in the fetal liver [29,30]. Developmental toxicity (increased fetal resorptions and decreased maternal weight gain) was determined to be surface charge-dependent after multiple exposures to Fe_2_O_3_-NPs during pregnancy, with positively charged polyethyleneimine-coated NPs (PEI-NPs) displaying greater toxicity and presence in the fetal liver over negatively charged poly(acrylic acid)-coated NPs (PAA-NPs) [29]. A study by Noori *et al.* examining the developmental toxicity of dimercaptosuccinic acid (DMSA)-coated Fe_3_O_4_-NPs, observed that a single *in utero* exposure to iron oxide NPs (≥100 mg/kg) on gestation day (GD) 8 led to a significant increase in pup mortality (~70%), a decrease in offspring weights, and a decrease in offspring testicular germ cells at postnatal day (PND) 50 [30]. No effects were observed in either study with lower single doses of NPs (10 or 50 mg/kg, respectively) or with eight consecutive doses of 10 mg/kg/day [29,30].

The experiments described herein were designed to further elucidate the influence of surface charge and dose on the developmental toxicity previously observed by our lab [29]. Experiments described herein were designed to determine whether a single, low or high dose of surface charged iron oxide NPs, given at a particular gestational period, will result in developmental toxicity [29]. Animals were given a single low (10 mg/kg) or high (100 mg/kg) dose of NPs during a period of major organogenesis (GD 8, 9, or 10). The influence of surface charge on toxicity was examined due to its previously observed effects on NP toxicity, where positively-charged NPs were observed to be more fetotoxic in one study [29], while increased offspring mortality and germ cell loss were observed with negatively-charged NPs in another [30]. The results of this investigation provide more insights into the role of surface charge and dose on the short- and long-term developmental toxicity of iron oxide NPs, which may lead to the safer design of future engineered nanomaterials. No developmental toxicity was observed after a single, low (10 mg/kg) dose of either positively- or negatively-charged NPs. However, a single, high dose (100 mg/kg) of either positively- or negatively-charged NPs resulted in short- and long-term developmental toxicity. Severity of the effects was dependent both on NP surface-charge and timing of exposure, with positively-charged NPs inducing greater toxicity later during organogenesis (GD 9 or 10) and negatively-charged NPs inducing more mild toxicity when exposed earlier during organogenesis (GD 8).

## 2. Results

### 2.1. Maternal Evaluations

No signs of morbidity, mortality or other clinical signs of toxicity (e.g., changes in appearance or behavior) were observed in the dams of any treatment groups, including the non-treated controls, before or during gestation. The overall conception rates were similar between treatment groups (71%–100%) (Table 1). Non-pregnancies were considered unrelated to treatment as they were present in comparable numbers across treatments, including control animals, and did not significantly affect conception rates (Table 1). 

**Table 1:** Maternal parameters of mating and fertility for mice treated with 10 mg/kg (**A**) or 100 mg/kg (**B**) of iron oxide NPs during gestation.

(**A**)	**Low Dose (10 mg/kg Body Mass)**						
	**Control**	**PEI8+**	**PEI9+**	**PEI10+**	**PAA8−**	**PAA9−**	**PAA10−**
Mated females, *n*	42	16	16	15	15	15	15
Pregnant dams, *n*	40	15	15	14	12	15	15
Non-pregnant, *n*	2	1	1	1	3	0	0
Conception rate, *%*	95	94	94	93	80	100	100
Dam mortality, *n*	0	0	0	0	0	0	0
(**B**)	**High Dose (100 mg/kg Body Mass)**						
	**Control**	**PEI8+**	**PEI9+**	**PEI10+**	**PAA8−**	**PAA9−**	**PAA10−**
Mated females, *n*	42	14	11	11	12	12	13
Pregnant dams, *n*	33	10	9	9	12	10	11
Non-pregnant, *n*	9	4	2	2	0	2	2
Conception rate, *%*	79	71	82	82	100	83	85
Dam mortality, *n*	0	0	0	0	0	0	0

Maternal weight gain (MWG) during pregnancy was evaluated by subtracting the weight of the dam on GD 0 from the final weight of the dam minus the gravid uterus on GD 17. MWG is an indication of maternal health during gestation. Alterations in MWG indicate a problem with the mother’s health during pregnancy and can have long-term effects on the developing fetus [31,32]. MWGs were unaffected by any of the “low dose” or “high dose” treatment groups (10 mg/kg and 100 mg/kg, respectively), as seen on Table 2. It is noted, however, that a single, high dose of iron oxide NPs (100 mg/kg) given on GD 10, regardless of charge, results in an apparent decrease in MWG (Table 2), though this trend was not statistically significant. 

**Table 2:** Maternal weight gain (MWG) in g ± the standard error of the mean (*SEM*) during gestation of mice treated with 10 mg/kg (**A**) or 100 mg/kg (**B**) iron oxide NPs on various days of gestation (GD 8, 9, or 10). MWG was determined by subtracting the initial body mass of the dams on GD 0 from the body mass of the dams on the day of sacrifice (GD 17) minus the gravid uteri. No significant differences were observed between treatment groups and the control.

(**A**)	**Low Dose (10 mg/kg Body Mass)**						
	**Control**	**PEI8+**	**PEI9+**	**PEI10+**	**PAA8−**	**PAA9−**	**PAA10−**
MWG, g *± SEM*	9.4 ± 0.5	8.9 ± 0.5	10.0 ± 0.8	10.5 ± 0.9	8.0 ± 0.7	9.6 ± 0.5	10.7 ± 0.9
(**B**)	**High Dose (100 mg/kg Body Mass)**						
	**Control**	**PEI8+**	**PEI9+**	**PEI10+**	**PAA8−**	**PAA9−**	**PAA10−**
MWG, g *± SEM*	10.4 ± 0.7	11 ± 1	11 ± 1	8 ± 2	10 ± 1	10 ± 1	8 ± 2

### 2.2. Embryo-Fetal Cesarean Evaluations

Observations from the cesarean evaluations are summarized in Table 3. Dams were sacrificed on GD 17, prior to parturition in order to evaluate litter parameters such as resorptions, fetal weights, and gross or skeletal malformations. A sobering finding of this report is the significant increase in fetal resorptions observed after a single exposure to a high dose of PEI-NPs (Table 3). Fetal resorptions occur during gestation and are indicative of fetal death. Timing of embryo/fetal loss can be determined as “early”, resulting in the complete resorption of the embryo, or “late”, resulting in partial resorption and a more complete fetus. A high dose (100 mg/kg) of PEI-NPs given on GD 10 resulted in significantly increased fetal resorptions (*p* < 0.05), with several total litters lost (Table 3). This effect was not observed in litters dosed with PEI-NPs on GD 8 or 9, indicating a critical window of toxicity to the developing fetus. By GD 10, approximately one in four of the pups were lost in the treatment group exposed to the high dose of PEI-NPs (100 mg/kg). Resorption rates were also elevated for dams given a high dose of negatively-charged PAA-NPs on GD 8 (100 mg/kg) compared to the control (Table 3). The resorption rate for PAA8− was ~22%.

No significant increases in resorption rates were observed in the “low dose” in any treatment group (Table 3). Interestingly, low dose PEI9+ and PAA10− mice displayed slightly lower resorption rates than the control (*p* < 0.05 and *p* < 0.1, respectively), though compared to historical control values these results were not significant. It has been well established that iron is necessary for gestation and iron supplementation (at low levels) can be beneficial [33]. As expected from resorption rates, fetal viability was unaffected with exposure to a single low dose of either iron oxide NP, regardless of charge.

Body masses of live fetuses were examined for each group following cesarean sections. Slight, but significant decreases in fetal body masses (FBMs) were recorded for mice dosed with a high level (100 mg/kg) of PEI-NPs on GD 10 or PAA-NPs on GD 9 (Table 3). For the PEI10+ treatment group, which already displayed increased fetal death (42% resorptions) as mentioned above, the FBM of the surviving fetuses was significantly reduced (*p* < 0.05). FBM is an indicator of fetal health. Taken together, increased fetal death and decreased FBM (and possibly the trend toward lower MWG) observed after a single high exposure (100 mg/kg) on GD 10 clearly demonstrate the toxicity of positively-charged PEI-NPs during later stages of gestation. Decreased FBM observed with negatively-charged PAA-NPs on GD 9, however, was not co-incident with an increase in resorptions or alteration in MWG, indicating a different, less severe mechanism of developmental toxicity. This contrast between the apparent toxicity of PEI-NPs and PAA-NPs at large exposure levels during organogenesis provide further evidence for the sensitivity of NP developmental toxicity to surface charge. As expected, FBM was not altered by treatment with the low dose (10 mg/kg) of either iron oxide NP, regardless of charge (Table 3).

**Table 3:** Fetal parameters following cesarean on GD 17 for dams treated with 10 mg/kg (**A**) or 100 mg/kg (**B**) of iron oxide NPs on GD 8, 9, or 10. * Significant difference between treatment and control (*p* < 0.05). ^‡^ Trend between treatment and control at (*p* < 0.1).

(**A**)	**Low Dose (10 mg/kg Body Mass)**						
	**Control**	**PEI8+**	**PEI9+**	**PEI10+**	**PAA8−**	**PAA9−**	**PAA10−**
Dams/live fetuses, *n*/*n*	22/263	10/128	10/149	9/112	6/79	9/126	9/128
Litters with resorbed or dead fetuses *(n*/*%)*	10/43	4/40	1/10	3/33	3/50	2/22	1/11
Post-implantation loss, *% ± SEM*	5 ± 2	9 ± 4	1 ± 1 ^‡^	4 ± 2	5 ± 2	3 ± 2	1 ± 1 ^‡^
Total litter loss, *n/%*	0	0	0	0	0	0	0
Fetal body mass, (GD 17), g *± SEM*	1.00 ± 0.03	0.99 ± 0.03	0.99 ± 0.04	0.96 ± 0.05	0.99 ± 0.04	0.99 ± 0.03	0.94 ± 0.04
(**B**)	**High Dose (100 mg/kg Body Mass)**						
	**Control**	**PEI8+**	**PEI9+**	**PEI10+**	**PAA8−**	**PAA9−**	**PAA10−**
Dams/live fetuses, *n*/*n*	9/104	4/43	5/50	5/38	4/40	3/10	5/52
Litters with resorbed or dead fetuses *(n*/*%)*	2/22	1/25	0/0	3/60	3/75	2/67	3/60
Post-implantation loss, *% ± SEM*	1 ± 1	2 ± 1	0 ± 0	42 ± 23 *	22 ± 17 *	6 ± 4	5 ± 2
Total litter loss, *(n*/*%)*	0	0	0	2/40	0	0	1/20
Fetal body mass, (GD 17), g *± SEM*	1.01 ± 0.03	1.02 ± 0.02	1.02 ± 0.06	0.94 ± 0.02 *	1.00 ± 0.07	0.93 ± 0.01 *	1.01 ± 0.12

### 2.3. Fetal Morphology Evaluations

**Figure 1:**
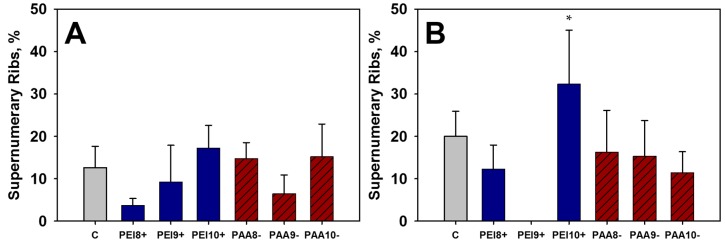
Supernumerary rib observations from fetuses of mice treated with 10 mg/kg (**A**) or 100 mg/kg (**B**) of iron oxide NPs and the control, C, on various days of gestation (GD 8, 9, or 10). * Significant differences between treatment group and the control (*p* < 0.05).

External evaluations were performed following cesarean sections for all treatments. No malformations or variations were observed for any treatment group given the low (10 mg/kg) dose of iron oxide NPs. A single incidence of hypoplasia of the tail was observed in the PEI9+ group given the high dose (100 mg/kg) of PEI-NPs on GD 9. No incidences of skeletal malformations were observed. Supernumerary, or accessory ribs, are common variations present even in the absence of treatment. Supernumerary ribs were observed and quantified for treated and control groups (Figure 1). No significant differences were observed in the incidence of supernumerary ribs or any other variations in the low dose treatment groups (10 mg/kg). However, in the high dose treatment groups (100 mg/kg) an increase in supernumerary ribs was observed in the PEI10+ (~30% incidence) treatment compared to the controls (~20% incidence). Increased incidence in supernumerary ribs is often utilized as an indicator of toxicity, though the significance of this endpoint is a subject of debate [34]. The functional relevance of an increased incidence of supernumerary ribs is currently unknown, though various factors may influence their formation including maternal stress, strain differences, and exposure to a toxicant during early pregnancy.

### 2.4. Offspring Evaluations

Remaining treated dams that were not sacrificed for cesarean examinations on GD 17 were allowed to give birth naturally. Body mass and general well-being (clinical signs) were monitored for the offspring of treated dams following parturition. Interestingly, a slight but significant (*p* < 0.05) increase in litter size was observed for dams treated with the low dose (10 mg/kg) of PEI- NPs on GD 9 (Table 4). In addition, trends of increased litter size were observed for PAA8− and PAA9− (*p* < 0.1). No significant alterations in litter size were observed for dams treated with 100 mg/kg of NPs.

**Figure 2:**
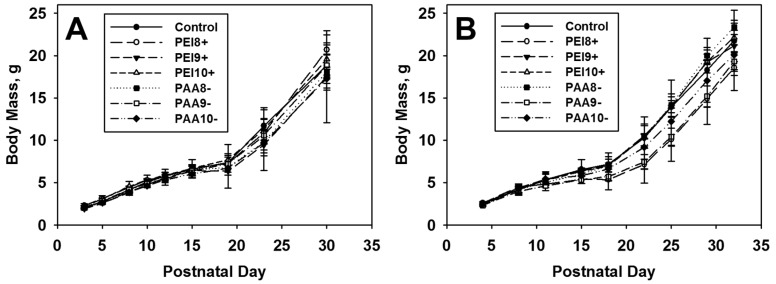
Growth charts of mice exposed to 10 mg/kg (**A**) or 100 mg/kg (**B**) of iron oxide NPs *in utero* on various days of gestation (GD 8, 9, or 10). Calculations include values from cesarean evaluations and dams that littered.

Pups were monitored through adulthood, after which those receiving 100 mg/kg were either mated or sacrificed and their reproductive organs collected as described below. No significant differences were observed in the offspring body mass compared to vehicle treated controls for any dosage level of iron oxide NPs (Figure 2). No increases in pup mortality were observed for any treatment group. 

**Table 4:** Litter parameters for offspring exposed to 10 mg/kg (**A**) or 100 mg/kg (**B**) of iron oxide NPs on GD 8, 9, or 10 *in utero*. Litter size represents results from cesarean evaluations and pups birthed naturally. * Significant differences between treatment and control (*p* < 0.05). Controls from both replicates were combined for litter size analyses as they underwent identical treatments. ^‡^ Trends between treatment and control at (*p* < 0.1).

(**A**)	**Low Dose (10 mg/kg Body Mass)**						
	**Control**	**PEI8+**	**PEI9+**	**PEI10+**	**PAA8−**	**PAA9−**	**PAA10−**
Dams/live pups, *n/n*	18/251	5/70	5/65	5/63	5/73	8/81	6/76
Litter size, mean *± SEM*	12.8 ± 0.4	13.2 ± 0.5	14.3 ± 0.4 *	12.5 ± 0.8	13.8 ± 0.6 ^‡^	13.8 ± 0.5 ^‡^	13.6 ± 0.4
(**B**)	**High Dose (100 mg/kg Body Mass)**						
	**Control**	**PEI8+**	**PEI9+**	**PEI10+**	**PAA8−**	**PAA9−**	**PAA10−**
Dams/live pups, *n/n*	24/317	6/80	4/49	4/55	8/97	7/97	6/84
Litter size, mean *± SEM*	12.8 ± 0.5	12 ± 1	11 ± 1	10 ± 2	11 ± 1	13.7 ± 0.7	12 ± 1

#### 2.4.1. Offspring Uterine Histopathology

**Figure 3:**
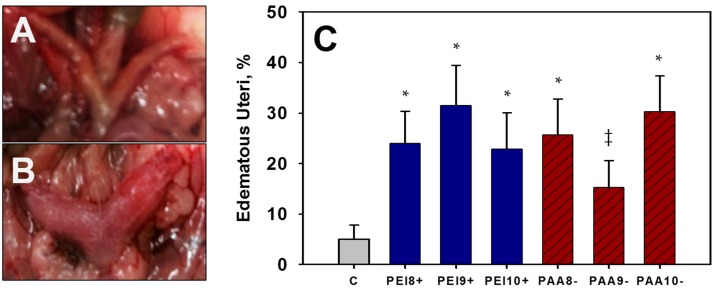
Representative picture of an observed typical uterus (**A**) or edematous uterus (EU) (**B**), as well as incidence of EU (**C**) observed for all treatment groups exposed to 100 mg/kg PEI-NPs or PAA-NPs *in utero*, and the control, C. * Significant difference between treatment group and the control (*p* < 0.05). ^‡^ Trend between treatment and control at (*p* < 0.1).

Adult female offspring of mice dosed with 100 mg/kg iron oxide NPs were sacrificed, and their uteri were collected for histological examination. During the uteri collection, edematous uteri (EU) were observed with a greater apparent frequency in adult mice treated with 100 mg/kg NPs *in utero* compared to control mice. Dramatic increases in the incidence of EU were observed in adult mice exposed to 100 mg/kg of either PEI-NPs or PAA-NPs compared to the control (*p* < 0.05) (Table 5, Figure 3). EU was observed in approximately 15%–31% of the pups (40%–54% of the litters) examined for each NP-treated group, and ~5% of the offspring (9% of the litters examined) in the control group. The cause of the EU is unknown, but a clear increase in incidence was observed in NP-exposed mice.

**Table 5:** Incidence of edematous uteri (EU) for offspring exposed to 100 mg/kg of iron oxide NPs on GD 8, 9, or 10 *in utero*. * Significant differences between treatment and control (*p* < 0.05). ^‡^ Trends between treatment and control at (*p* < 0.1).

	High Dose (100 mg/kg Body Mass)						
	Control	PEI8+	PEI9+	PEI10+	PAA8−	PAA9−	PAA10−
Littered/offspring, *n*	32/60	13/46	12/35	12/35	15/39	10/46	10/43
Edematous uteri, mean *± SEM*	5 ± 3	24 ± 6 *	31 ± 8 *	23 ± 7 *	26 ± 7 *	15 ± 5 ^‡^	30 ± 7 *
Litters affected, *n*/*%*	3/9	7/54	6/50	5/42	6/40	4/40	4/40

**Figure 4:**
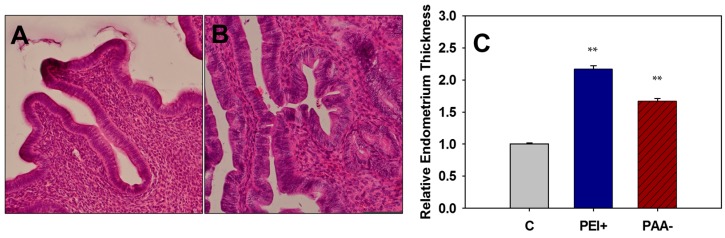
Hematoxylin and eosin stained uterine sections from adult control mice (**A**) or those exposed to 100 mg/kg of iron oxide NPs *in utero* (**B**) (magnification of **A**,**B**: 200×). Relative endometrial thicknesses (**C**) were measured for all treatment groups exposed to 100 mg/kg iron oxide NPs *in utero* and the control, C, with GD 8, 9, or 10 pooled for all analyses. ** Significant differences between treatment group and the control (*p* < 0.01).

To further investigate the uteri of mice exposed to iron oxide NPs *in utero*, uterine samples were sectioned, stained, and examined histologically. All of the uteri examined were in the proliferative stage of the uterine cycle, which occurs during proestrus of the estrous cycle. Mice exposed to iron oxide NPs *in utero* displayed a dramatic increase in endometrial thickness (endometrial hyperplasia), with increased preference toward pseudostratified columnar epithelium (as opposed to typical simple columnar) when compared to the control. A more pronounced hyperplasia was observed after exposure to positively-charged PEI-NPs than negatively-charged PAA-NPs (*p* < 0.01) (Figure 4). Endometrial thickness varies based on the stage of uterine cycle, however, as all mice examined were in the same stage, the expectations are that the endometrial thickness would be similar. Endometrial hyperplasia can be linked with prolonged proestrus (estrogen stimulated) or endometritis and is often a precursor to endometrial cancer [35,36]. Ovaries were also examined histologically, but lacked overt signs of toxicity.

#### 2.4.2. Offspring Testicular Histopathology

**Figure 5:**
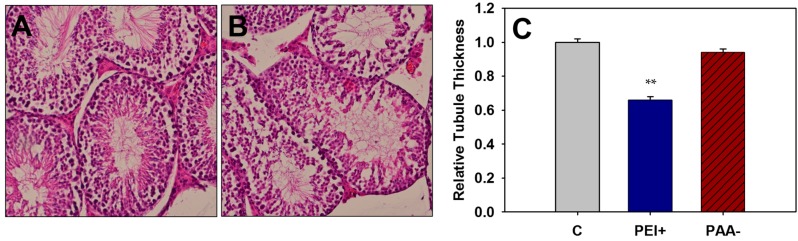
Hematoxylin and eosin stained testis sections from adult control mice (**A**) or those exposed to 100 mg/kg of iron oxide NPs *in utero* (**B**) (magnification of **A**,**B**: 200×). Relative germinal epithelial thicknesses of the seminiferous tubule (**C**) were measured for all treatment groups exposed to 100 mg/kg iron oxide NPs *in utero* and the control, C, with GD 8, 9, and 10 pooled for all analyses. ** Significant differences between treatment group and the control (*p* < 0.01).

Testes were collected from adult mice exposed to 100 mg/kg of iron oxide NPs *in utero*, for evaluation of the germinal epithelium. Seminiferous germinal epithelium thickness was measured as an indication of germ cell loss, and assessed for presence of spermatogonia, spermatocytes, and round spermatids. All GDs were combined per treatment for analysis. A significant decrease in tubule epithelium was observed for mice treated with a high dose (100 mg/kg) of positively-charged PEI-NPs compared to controls (*p* < 0.01) (Figure 5). Decreased germinal epithelium is indicative of germ cell loss or reduction and could be a direct result of exposure to NPs *in utero*. 

#### 2.4.3. Second Generation Fertility Evaluations

Adult offspring exposed to NPs *in utero* were mated with control mice in order to assess whether the observed testicular or uterine histopathology influenced the fertility of mice treated with 100 mg/kg of positively- or negatively-charged NPs. Specifically, the influence of increased uterine endometrium with increased EU and the decreased germinal epithelium and tubule diameter was examined by mating treated mice to control, untreated mice. Fertility data are presented in Table 6.

**Table 6:** Fertility indices for offspring exposed to 100 mg/kg of iron oxide NPs on GD 8, 9, or 10 *in utero*. Each treated mouse was mated to a control. * Significant difference between treatment group and the control (*p* < 0.05). ^‡^ Trend between treatment and control at (*p* < 0.1).

	High Dose (100 mg/kg Body Mass)						
	Control	PEI8+	PEI9+	PEI10+	PAA8−	PAA9−	PAA10−
Mated females, *n*	13	19	12	14	17	19	13
Pregnant dams, *n*	13	17	12	13	17	18	12
Non-pregnant, *n*	0	2	0	1	0	1	1
Conception rate, *%*	100	89	100	93	100	95	92
Litters/live fetuses, *n*	13/162	17/204	12/139	13/157	17/237	18/234	12/145
Fetal loss, *% ± SEM*	3 ± 1	11 ± 6	21 ± 11 *	13 ± 7 ^‡^	5 ± 2	5 ± 2	12 ± 8
Total litter loss, (*n*/*%)*	0	1/6	2/17	1/8	0	0	1/8
Litter size, mean *± SEM*	12.5 ± 0.9	12 ± 1	12 ± 2	12 ± 1	13.9 ± 0.6	13.0 ± 0.7	12 ± 1

**Figure 6:**
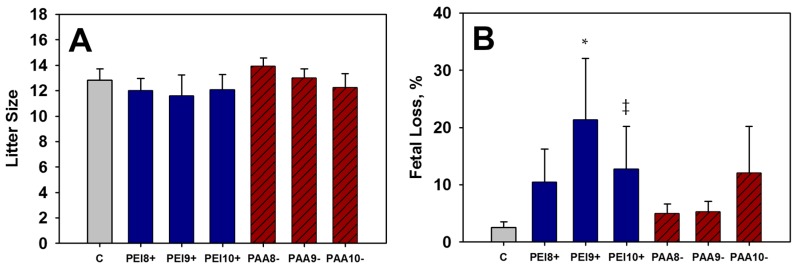
Litter size (**A**) and fetal loss (**B**) measured from matings of adult mice previously exposed to 100 mg/kg of iron oxide NPs *in utero*, and the control, C, on various days of gestation (GD 8, 9, or 10). * Significant difference between treatment group and the control (*p* < 0.05). ^‡^ Trend between treatment and control at (*p* < 0.1).

Treated mice, regardless of treatment group were able to conceive successfully. Five mice did not become pregnant after mating in different treatment groups. Non-pregnancies were not considered significant as they were all in different treatment groups, and the conception rates remained similar. Litter sizes (number of live fetuses or pups per litter) were unaffected by *in utero* exposure to iron oxide NPs, regardless of charge (Table 6, Figure 6A). However, increased fetal loss was observed throughout the course of cesarean evaluations for mice treated with positively-charged PEI-NPs on GD 9 or 10 *in utero* (Figure 6B). In addition, 10.5% of mice treated with PEI-NPs *in utero* lost entire litters, contributing to the elevated resorption levels observed. 

Though increased resorptions were observed in groups treated with 100 mg/kg PEI-NPs *in utero*, no apparent differences in litter sizes were observed, nor in the ability of the mice to become pregnant. It has been shown previously that it is difficult to knock down fertility in mice. In humans for example, <15–20 million sperm/mL is considered subfertile due to oligospermia (low sperm count), with normal values ranging from 20–200 million sperm per mL [37]. A two-fold decrease in sperm production is enough to begin to see infertility in humans [38]. In rodents, however, a ~20-fold reduction in sperm (about 5% of normal counts) is required to begin to see any fertility changes [38]. Increased fetal deaths and total litter losses for second generation mating were observed when either the male or female was exposed to a high dose (100 mg/kg) of positively-charged iron oxide NPs *in utero*.

## 3. Discussion

The study described herein provides more substantial insights into the developmental and reproductive toxicity of iron oxide NPs by examining the influence of both surface charge (surface coating) and dose during critical windows of major organogenesis (GD 8, 9, or 10). Briefly, pregnant dams were dosed with a single *i.p.* “low” or “high” dose of positively-charged PEI-NPs or negatively-charged PAA-NPs (28–30 nm) on GD 8, 9, or 10. Dams and fetuses/pups were evaluated for signs of developmental toxicity. Additionally, the offspring of dams dosed with NPs were monitored and their reproductive capacity evaluated by histopathology and fertility testing. For clarity, the discussion below is arranged by treatment level (low or high).

### 3.1. “Low Dose” of Iron Oxide NPs (10 mg/kg)

Previously reported studies have investigated the developmental toxicity of iron oxide NPs in mammals utilizing high doses [30], multiple low doses [29], or continuous infusions of NPs throughout gestation [3,39]. A single, low dose (10 mg/kg NPs or ~2.5 mg/kg Fe) was chosen to investigate the impact of a biomedically-relevant dose of iron oxide NPs given during gestation. Overall, no negative effects were observed for any treatment group, regardless of surface-charge or window of exposure (GD 8, 9, or 10). Interestingly, several positive trends were observed in several treatment groups, which may be due to a supplementary effect of iron, which is necessary for both the developing fetus and the pregnant dam [33].

No increased incidence of dam mortality or toxicity was observed, including no decreases in MWG. This is consistent with previous studies examining a single dose of PEI-NPs or PAA-NPs given on GD 9 [29]. Interestingly, a slight, yet not significant, increase in MWG was observed for dams treated with either iron oxide NP given on GD 10 (PEI10+, PAA10−), indicating a potential positive effect of supplementation. Iron is a routine supplement for pregnant women, as it is necessary for both the mother and the developing fetus [33]. The World Health Organization (WHO) estimated that 41.8% of pregnant women across the globe are anemic, with at least half of these cases due to iron deficiency [33]. Therefore, it is not entirely unexpected that supplemental iron may be beneficial to pregnant women. This effect was only observed with the latest dose (GD 10), possibly due to the relative dose received by the dam. Mice were dosed based on maternal body mass 10 mg NPs per kg of body mass. As the fetuses grow inside the dam (~10–15/litter), their body mass added to the total dose received, resulting in a larger load of NPs given to the mother.

Fetal litter parameters were largely unchanged compared to controls for all NP treatment groups across GDs. FBM was consistent between treatments and the control. No increases in fetal death (resorptions), including total litter losses, were observed with any treatment, consistent with previous findings dosing on GD 9 [29]. However, a trend toward decreased fetal death (increased fetal survival) was observed for PEI9+ and PAA10− treated litters (*p* < 0.1). No gross or skeletal malformations were observed for either controls or iron oxide NP-treated mice. Supernumerary ribs, a spontaneous variation in rodents, were observed in the treated and control fetuses with similar frequencies [40]. 

Litter sizes were determined as the number of live fetuses or pups, and quantified for each treatment. No decreases in litter sizes were observed for any treatment. Dams receiving positively-charged PEI-NPs on GD 9 (PEI9+) actually had significantly larger litter sizes (*p* < 0.05) than the control. Additionally, two of the treatment groups receiving 10 mg/kg negatively-charged PAA-NPs trended higher in litter size than the control (*p* < 0.1). Offspring growth was identical between treatments following birth and began to diverge at the onset of puberty due to differing sex ratios between treatment groups. Offspring body mass and growth rates are common indicators of overall health. No indications of altered pup weights were observed for any treatment compared to control mice. 

Overall, no signs of toxicity were observed after a single, low dose of iron oxide NPs, regardless of charge. Maternal and fetal health was not negatively affected by a single 10 mg/kg dose of iron oxide NPs. In fact, it appears that a “low” dose of iron oxide NPs may provide some benefit to the mother and developing fetus. Previously, multiple low doses (10 mg/kg) of identical iron oxide NPs (PEI- or PAA-coated) were given to pregnant mice on eight consecutive days of gestation (GD 9–16) resulting in the passage of PEI-NPs across the placenta and into the fetal liver [29]. Multiple exposures also led to increased fetal death after exposure to either negatively- or positively-charged NPs, with greater fetal loss observed with the latter [29]. Unfortunately, as one exposure during a critical period of organogenesis appears benign or possibly beneficial to fetal development, continued exposure even at this low dose, results in accumulation and fetal death with positively-charged PEI-NPs.

### 3.2. “High Dose” of Iron Oxide NPs (100 mg/kg)

In order to assess the influence of dose and surface charge on the reproductive and developmental toxicity of iron oxide NPs during critical windows of organogenesis, pregnant dams were given a single, high dose (100 mg/kg NPs or 25 mg/kg Fe) of surface-charged iron oxide NPs. Fetuses and pups of the exposed mice were examined and weighed to determine developmental toxicity and teratogenicity. Previously, alterations in testis histology including loss of mature sperm, spermatids, primary spermatocytes, and round spermatids were observed after high doses (>50 mg/kg) of DMSA-coated Fe_3_O_4_-NPs (3–9 nm) [30]. Therefore, the reproductive organs of pups exposed to iron oxide NPs *in utero* were assessed histologically for similar pathology. Offspring of dosed dams (exposed *in utero*) were mated to control mice in order to assess their reproductive capacity.

No morbidity, mortality, or other clinical signs of toxicity were observed for dams treated with high doses (100 mg/kg) of either positively- or negatively-charged iron oxide NPs. This is consistent with previous research, which exposed rats to even higher doses of dextran-coated iron oxide NPs, given over multiple days of gestation [39]. Slight decreases in MWG were observed for animals treated with NPs on GD 10, regardless of charge, though this difference was not significant. Previous studies has also noted a decrease in maternal weight gain after high cumulative exposures to iron oxide NPs, including a significant decrease in MWG observed after mice were dosed with identical PEI-NPs at 10 mg/kg for 8 consecutive days of gestation [29,39]. Taken together, these results indicate that the deleterious effects on maternal health were likely due to the accumulation of NPs after several “low” doses of PEI-NPs [29].

Increased fetal death was observed in a few, but not all, treatments receiving 100 mg/kg iron oxide NPs, and appear to depend on the stage of gestation. Positively-charged PEI-NPs induced the largest increase in resorptions/fetal death with approximately 42% loss after exposure on GD 10 (*p* < 0.05). These results are in agreement with the previously reported ~22% resorption rate observed when mice were given 8 consecutive low doses (10 mg/kg) of PEI-NPs [29]. Negatively-charged PAA-NPs also induced increased resorptions, though not to the extent of PEI-NPs, and not on the same day. PAA-NPs given earlier in gestation (on GD 8) resulted in ~22% resorptions (*p* < 0.05). Previously, ~14% resorptions were reported for mice given 10 mg/kg of PAA-NPs for 8 consecutive days [29]. Several total litters were lost after treatment resulting in two complete early resorptions for PEI10+ and one for PAA10−. Taken together, these results indicate a mechanism of toxicity for a high dose of positively-charged iron oxide NPs leading to fetal loss beginning on GD 10.

In addition to an increased observation of resorptions, PEI-10+ FBMs were significantly lower (*p* < 0.05) on GD 17 than the control, indicating potential fetotoxicity. FBM was also reduced (*p* < 0.05) for the litters exposed to PAA-NPs on GD 9, though no increases in resorptions were observed for this treatment. Previously, no alterations in FBM were observed after eight consecutive low doses of PEI- or PAA-NPs [29]. To put these results in perspective, the high dose exhibiting increased resorptions (100 or 25 mg/kg of iron) is much higher than one would expect to be exposed to for a single biomedical purpose. Though the NPs share the same Fe_2_O_3_ core and similar hydrodynamic sizes (28–30 nm), the applied surface coatings significantly alter their mechanisms of developmental toxicity. Positively-charged PEI-NPs have been shown to accumulate to a greater extent in fetal tissues and display an elevated risk for fetal death with high doses or multiple low doses given during pregnancy, especially at later points of gestational progression [29]. Negatively-charged PAA-NPs, on the other hand, did not accumulate in the developing fetus after multiple exposures, but still display elevated fetal death with high doses given early in gestation and after multiple exposures during pregnancy [29].

No differences were observed in litter sizes (including both cesarean and littered dams). Of the surviving fetuses, a single incidence of tail hypoplasia (missing tail) was observed in the PEI9+ treatment group, with no further apparent external malformations observed for any other treatment group. A single incidence of tail hypoplasia has been previously observed when mice were treated orally with 100 mg/kg/day of zinc oxide NPs, but was not attributed to treatment [40]. No skeletal malformations were observed for any treatment group. An increased incidence of supernumerary ribs was observed in fetuses in the PEI10+ treatment group (*p* < 0.05). This variation occurs spontaneously in rodents, with increased incidence observed in response to certain toxicants, though the mechanism and significance remains uncertain [40]. Growth rates remained similar between treatments groups, with no significant increase in mortality observed. This contrasts with previous literature, which observed ~70% pup mortality by adulthood after acute NP exposure [30].

The reproductive capacity of mice exposed to iron oxide NPs *in utero* was determined by mating a second generation of offspring from mice exposed to NPs *in utero* with control animals as well as examining the histopathology of their reproductive organs (uterus, ovary, and testis). During the course of collecting female reproductive organs from treated animals, an increased incidence of EU was observed, then quantified. Significant increases (*p* < 0.05) in the incidence of EU were observed in almost all treatment groups compared to the control, regardless of charge, with the exception of PAA9−, which still displayed an upward trend (*p* < 0.1). Subsequently, the endometrial linings of the uteri of treated and control mice were measured, and a significant increase in endometrial thickness (endometrial hyperplasia) was observed for both PEI- and PAA-NP-treated groups. The magnitude of the change was greater for positively-charged PEI-NP treated mice. In addition to endometrial hyperplasia and EU, alterations in the endometrial linings were noted for NP-treated mice. An apparent shift toward pseudostratified columnar epithelium instead of the typical simple columnar epithelium of the uterine endometrium was observed for mice treated with 100 mg/kg iron oxide NPs, regardless of charge. This result may be due to excessive proliferation, induced by NP exposure and has been identified as a precursor to carcinoma [35]. 

Testis sections from mice exposed to a high dose (100 mg/kg) of iron oxide NPs were also examined for histopathological alterations. Previously, mice exposed to a single, high dose (50, 100, 200, or 300 mg/kg) of DMSA-coated iron oxide NPs (3–9 nm) *in utero* presented with seminiferous tubule degeneration marked by decreased spermatogonia, primary spermatocytes, round spermatids, and elongated spermatozoa [30]. Seminiferous tubules of adult mice exposed to PEI- or PAA-NPs *in utero* were examined and the germinal epithelium measured. The germinal epithelium was found to be significantly reduced in the positively-dosed PEI-NP exposed groups (100 mg/kg), indicating a loss of germ cells. An apparent decreased in the numbers of spermatogonia was also noted for treated mice, regardless of charge or dosage day. It has been shown previously that PEI-NPs can efficiently cross the placenta and accumulate in the developing fetus [29]. The presence of NPs in the developing fetus may alter testicular development. Apparent decreased numbers of spermatogonia were observed for mice dosed with 100 mg/kg iron oxide NPs *in utero*. Noori *et al.* observed decreased numbers of spermatogonia, primary spermatocytes, spermatids, and mature spermatozoa of adult mice exposed to DMSA-coated iron oxide NPs on GD 8 (>50 mg/kg) [30].

None of the treatment groups receiving 100 mg/kg of either iron oxide NP displayed any difficulty mating and producing viable offspring. Conception rates remained around 90%–100%. However, increased incidence of fetal death was observed in the second generational study. Mice that were exposed to 100 mg/kg of iron oxide NPs *in utero* were mated to control mice, and examined prior to birth to determine the number of live *versus* dead fetuses. Again, litter sizes were unaffected by treatment, yet a significant increase in fetal deaths was observed for the treatment groups that received 100 mg/kg of positively-charged iron oxide NPs *in utero* (PEI-NPs) on GD 9 (21%) or GD 10 (13%). This observation is evidence that fetal exposure to iron oxide NPs (especially positively-charged NPs) not only influences the mother’s ability to produce viable offspring, but can also influence the reproductive capacity/fertility of the offspring. Further investigations are needed to elucidate the mechanisms behind the observed long-term developmental effects of positively-charged iron oxide NPs. Positively-charged NPs have already been shown to enter fetal tissues to a greater extent than similar negatively-charged NPs, but how the NPs are effecting development and reproduction are yet to be determined.

## 4. Materials and Methods

### 4.1. Iron Oxide Nanoparticles

Polyethyleneimine-coated (PEI-NPs) and poly(acrylic acid)-coated (PAA-NPs) iron oxide NPs were synthesized and characterized as previously described [29,41]. PAA is considered to be essentially non-toxic while the toxicity of PEI is dependent on molecular weight. Low molecular weight PEI (10 kDa) was utilized in this study due to its low cytotoxicity [42,43]. Both PEI-NPs and PAA-NPs did not form aggregates based on dynamic light scattering analyses performed as described previously. For in depth information on the synthesis and characterization of these NPs, see Di Bona 2014 and Xu 2011 [29,41]. The PEI-NPs carry a positive surface charge and have an average functionalized diameter of 28 nm, while the PAA-NPs carry a negative surface charge and have an average diameter of 30 nm. NPs were dispersed in sterile milliq-H_2_O (18 mΩ) prior to administration at 1 mg/mL, which allowed for a final dosage volume of 0.01 mL/g body mass.

### 4.2. Animals and Husbandry

Male and female Crl:CD1(ICR) (CD-1) mice were purchased from Charles River Breeding Laboratories (Wilmington, MA, USA) and acclimated for two weeks prior to experiments. CD-1 mice have large litters (average 14 pups), breed easily, and have ample reference data. Animals were maintained in the AAALAC-approved (Association for Assessment and Accreditation of Laboratory Animal Care International) Animal Care facility at The University of Alabama at 22 ± 2 °C with 40%–60% relative humidity and a 12 h photoperiod. Standard rodent diet (Teklad LM-485, Harlan, Madison, WI, USA) and tap water were provided *ad libitum* throughout the study.

After the initial period of acclimation, two untreated female mice were bred naturally with one male (2:1). Mating checks were performed twice daily. Confirmation of mating was achieved through the observation of a copulation plug, indicating GD 0. Immediately after conformation of mating, females were randomly assigned into treatment groups and individually housed in polycarbonate shoebox-style housing with hardwood bedding (29 cm × 19 cm × 13 cm). After completion of mating, male mice were euthanized using CO_2_ asphyxiation. All procedures and protocols were reviewed and approved by The University of Alabama’s Institutional Animal Care and Use Committee (IACUC) [Project Identification Codes: 11-363-1 (approved: 5 August 2011) and 12-369-1 (approved: 26 January 2012)] and in accordance with established guidelines from the International Conference of Harmonisation (ICH) and the *AVMA Guidelines for the Euthanasia of Animals* [44,45].

### 4.3. Treatments

After mating, female CD-1 mice were randomly assigned to the following treatment groups (Table 7). 

**Table 7:** Treatment groups received PEI- or PAA-coated iron oxide NPs at 10 or 100 mg/kg body mass or the vehicle control (H_2_O) on GD 8, 9, or 10.

NPs	Concentration	GD	Abbreviation	Group Number
PEI(+)	10 mg/kg (Low dose)	8	PEI8+	1
		9	PEI9+	2
		10	PEI10+	3
	100 mg/kg (High dose)	8	PEI8+	7
		9	PEI9+	8
		10	PEI8+	9
PAA(−)	10 mg/kg (Low dose)	8	PAA8−	4
		9	PAA9−	5
		10	PAA10−	6
	100 mg/kg (High dose)	8	PAA8−	10
		9	PAA9−	11
		10	PAA10−	12
Control H_2_O	0.01 mL/g	8, 9, or 10	C	13,14, and 15

Dams were treated by intraperitoneal (*i.p.*) injection of the appropriate dose of NPs in sterile milliq-H_2_O or vehicle control only (H_2_O) at a volume of 0.01 mL/g body mass on either GD 8, 9, or 10. The selected doses were based on a previous repeated-dose study conducted by our lab designed to elucidate the contribution of dose and surface charge on developmental toxicity [29]. The “low dose” (10 mg/kg) is equivalent to ~2.5 mg/kg iron and was chosen to represent the amount one might receive from a single biomedical use or exposure event [13]. The high dose (100 mg/kg) was chosen to directly compare with the effects observed previously after multiple low doses of NPs and examine if exposure on a specific GD correlates with toxicity [29]. Dosing was performed on days of major organogenesis to determine if the previously observed toxicity was a result of a single low dose on a specific GD or the result of an accumulation of NPs after 8 consecutive exposures (80 mg/kg, total). This dosage scheme also allows for comparison with the results observed by Noori *et al.*, who observed alterations in testis histology and growth of offspring dosed with iron oxide NPs *in utero* (≥100 mg/kg) [29,30]. Vehicle control groups (H_2_O only) were found to be statistically equivalent to each other and in agreement with historical data (unpublished results) regardless of treatment day (GD 8, 9, or 10). Therefore, control groups (13, 14, and 15) were subsequently pooled and presented as a single group referred to as “control” for each dosage group hereafter.

### 4.4. Observation of Dams and Cesareans

Dams were monitored throughout gestation for clinical signs of toxicity (e.g., morbidity, mortality, behavioral changes, and alterations in general appearance). Maternal body weights were measured on GD 0 and on the day of dosing (GD 8, 9, or 10). Approximately half of the dams were sacrificed one day prior to parturition on GD 17 by CO_2_ asphyxiation. Cesarean sections were performed on sacrificed dams by exposing and removing gravid uteri. Maternal body weights were measured (minus gravid uteri). Maternal weight gains were obtained by subtracting the weights obtained on GD 0 from the weights obtained on GD 17 minus the gravid uteri. Uteri were examined for numbers of live fetuses, early, and late resorptions to determine post-implantation loss. Early resorptions were identified as a dark black/brown spot resembling a blood clot while late resorptions were identified by the appearance of embryonic and placental tissue and/or fetal features.

### 4.5. Fetal Evaluations

Live fetuses were weighed individually and externally examined for gross malformations or variations (e.g., cleft palate or bent tail). Following gross examination, fetuses were euthanized by *i.p.* administration of Euthasol and fixed in 70% ethanol for skeletal evaluations. Following fixation, fetuses were subsequently eviscerated, cleared, and double-stained for bone and cartilage with Alizarin red and Alcian blue, respectively (Sigma-Aldrich, St. Louis, MO, USA) as previously described [46]. Skeletal examinations were performed on all fetuses for malformations and variations (e.g., split cervical arch, supernumerary ribs).

### 4.6. Offspring Evaluations

To examine the long-term effects of a single *in utero* exposure (100 mg/kg) to iron oxide NPs, remaining dams (approximately half of original numbers) gave birth naturally on GD 18. Litter sizes were recorded and the body masses of the offspring were recorded twice weekly, through adulthood. Adult mice were mated to controls for fertility analysis, or sacrificed for histological evaluation of their reproductive organs (ovaries, uteri, testes).

#### 4.6.1. Histopathology

Uteri, ovaries, and testes were collected from adult offspring (exposed to NPs *in utero*). Reproductive organs were fixed in 4% paraformaldehyde, embedded in paraffin, sectioned to 10 μm thickness, and mounted on glass microscope slides. After dehydration by a series of ethanols, the tissues were stained with hematoxylin and eosin (H&E) and examined via bright field light microscopy on a Zeiss Axioskop 40 FL microscope (Zeiss, Thornwood, NY, USA). Morphological alterations in seminiferous tubule and uterine epithelium histology were examined visually. Epithelial linings of the uteri and seminiferous tubules were quantified using ImageJ software (National Institutes of Health, Bethesda, MD, USA). Endometrium and seminiferous tubule measurements were chosen at random and measured from 6 uterine or testicular samples for the “high dose” (100 mg/kg) NP treated groups and the controls for a total of 100 measurements per treatment.

#### 4.6.2. Fertility

In order to assess if the reproductive capabilities of the mice exposed to 100 mg/kg iron oxide NPs *in utero* were affected by treatment, adult offspring of treated dams were mated with control mice at approximately 6 weeks of age. Mating was monitored as described above. Second generation ability to become pregnant and produce viable offspring were recorded for each treatment group. Female mice were sacrificed on GD 17 and numbers of live and dead fetuses were recorded to assess fetal loss and litter size.

### 4.7. Statistical Analysis

The litter or a pregnant dam were used as the experimental units for statistical analyses. Statistical analyses were performed using SPSS version 19.0 (SPSS, Inc., Chicago, IL, USA) or R statistical software version 3.2.1 (R Foundation for Statistical Computing, Vienna, Austria). Multiple replicates were performed, with data from each replicate calculated independently, tested for homogeneity of variance by Levene’s statistic, pooled, and analyzed to give the results reported. Parametric and non-parametric data were analyzed via one-way analysis of variance (ANOVA) or Kruskal–Wallis one-way ANOVA followed by a least significant difference (LSD) or Dunn’s *post hoc* test, respectively, to determine statistical significance (*p* < 0.05). Student’s *t*-tests and Mann–Whitney *U* tests were also employed when appropriate. Control groups receiving H_2_O on GD 8, 9, or 10 were found to be statistically equivalent per replicate to each other as well as to historical lab data for all parameters investigated and were therefore pooled and are represented together throughout this report. 

## 5. Conclusions

At low dosage levels (10 mg/kg), no maternotoxicity or fetotoxicity were observed with any treatment groups. Instead, a few positive effects such as decreased fetal death and increased litter size were observed for some treatment groups, possibly due to the benefits of supplemental iron during pregnancy. However, developmental toxicity was observed after a single, high (100 mg/kg) exposure to iron oxide NPs *in utero* and found to be and charge-dependent. A high dose (100 mg/kg) of iron oxide NPs increased fetal death when given later in gestation (GD 10) for positively-charged PEI-NPs and earlier in gestation (GD 8) for negatively-charged PAA-NPs, with greater losses occurring after exposure to the positively-charged PEI-NPs. Decreased FBM was also noted for the negatively-charged PAA-NPs given on GD 9. In addition to the fetotoxicity observed after the initial NP exposure for PEI10+, PAA8−, and PAA9−, secondary developmental effects were also evident that had long-lasting consequences. Regardless of the timing of exposure, all treatment groups receiving 100 mg/kg of positively-charged iron oxide NPs *in utero* displayed altered histology of the uterus or testis, resulting in excessive thickening of the endometrium and germ cell loss, respectively. These alterations did not affect conception rates, as all mice were able to mate and become pregnant, regardless of treatment (~90%–100%). However, a significant increase in fetal death was observed for treatments exposed to positively-charged PEI-NPs on GD 9 (21%) or GD 10 (13%). These results taken together indicate that a high dose of positively-charged iron oxide NPs given during later organogenesis, is more fetotoxic than an equivalent dose of negatively-charged iron oxide NPs. The PEI10+ treatment group in particular consistently displayed high developmental toxicity compared to other treatments. Future work is needed to expand upon the findings of this report and examine several more surface coatings with either positive or negative surface charge in order to definitively indicate surface charge as the primary modifier of the observed toxicity.
